# Effects of Ferulic Acid and γ-Oryzanol on High-Fat and High-Fructose Diet-Induced Metabolic Syndrome in Rats

**DOI:** 10.1371/journal.pone.0118135

**Published:** 2015-02-03

**Authors:** Ou Wang, Jia Liu, Qian Cheng, Xiaoxuan Guo, Yong Wang, Liang Zhao, Feng Zhou, Baoping Ji

**Affiliations:** 1 College of Food Science & Nutritional Engineering, China Agricultural University, Beijing, China; 2 China National Research Institute of Food & Fermentation Industries, Beijing, China; INRA, FRANCE

## Abstract

**Background:**

The high morbidity of metabolic dysfunction diseases has heightened interest in seeking natural and safe compounds to maintain optimal health. γ-Oryzanol (OZ), the ferulic acid (FA) ester with phytosterols, mainly present in rice bran has been shown to improve markers of metabolic syndrome. This study investigates the effects of FA and OZ on alleviating high-fat and high-fructose diet (HFFD)-induced metabolic syndrome parameters.

**Methods:**

Male SD rats were fed with a regular rodent diet, HFFD, or HFFD supplemented with 0.05% FA or 0.16% OZ (equimolar concentrations) for 13 weeks. Food intake, organ indices, serum lipid profiles, glucose metabolism, insulin resistance (IR) index and cytokine levels were analyzed. The mechanisms were further investigated in oleic acid-stimulated HepG2 cells by analyzing triglyceride (TG) content and lipogenesis-related gene expressions.

**Results:**

In the *in vivo* study, FA and OZ exhibited similar effects in alleviating HFFD-induced obesity, hyperlipidemia, hyperglycemia, and IR. However, only OZ treatment significantly decreased liver index and hepatic TG content, lowered serum levels of C-reactive protein and IL-6, and increased serum concentration of adiponectin. In the *in vitro* assay, only OZ administration significantly inhibited intracellular TG accumulation and down-regulated expression of stearoyl coenzyme-A desaturase-1, which might facilitate OZ to enhance its hepatoprotective effect.

**Conclusion:**

OZ is more effective than FA in inhibiting hepatic fat accumulation and inflammation. Thus, FA and OZ could be used as dietary supplements to alleviate the deleterious effects of HFFD.

## Introduction

Metabolic syndrome (MS) is a cluster of metabolic dysfunctions that includes hyperglycemia, hypercholesterolemia, hypertriglyceridemia and insulin resistance (IR), and accompanies type 2 diabetes mellitus, obesity and cardiovascular diseases. [1,2]. Approximately one-fourth of adults worldwide have been diagnosed with MS [3]. The high morbidity of MS can be partly attributed to modern dietary patterns. For example, diets rich in fats and sugars are the main culprits leading to MS symptoms [4, 5]. Among the common dietary sugars, fructose has been widely used in the production of beverages and sweets due to its low cost and preferred taste [6]. However, dietary fructose is almost totally absorbed and metabolized rapidly by the liver, undergoing a markedly different metabolic fate from glucose metabolism, resulting in deleterious effects, such as IR, obesity and hyperuricemia [7]. We have previously shown that fructose worsens the adverse effects of dietary fats on serum glucose and lipids regulation [8]. Therefore, there is a heightened interest in identifying highly effective compounds that lessen the health-threatening effects of fructose and fats while maintaining food palatability.

Phenolic acids and their derivatives are natural compounds that possess pharmacological properties. Ferulic acid (FA), a well-known natural antioxidant present in common fruits and vegetables, has been shown to improve glucose tolerance and lipid metabolism [9, 10]. Similarly, γ-oryzanol (OZ), the FA esters with phytosterols, mainly present in rice bran, exhibits numerous nutritive effects, such as free radical scavenging activity, anti-ulcer and neuromodulation [11,12]. Both FA and OZ demonstrated similar effects in preventing alcohol-induced liver injury and regulating glucose and lipid metabolism [13–15]. However, previous studies have used these compounds at similar mass fraction in the diets without considering their varying molecular weights and therefore their potential differential effects [13–15]. Furthermore, whether FA or OZ can be used as dietary supplements to improve parameters of MS is not known. In previous studies, the effects of these compounds on metabolic dysregulations were mostly evaluated under high fat feeding regimen. Given the reported adverse effects of fructose on metabolic balance, such as hepatic damage, dyslipidemia and IR [16, 17], and that the typical western diet contains a mixture of high fat and high fructose, it is important to examine the effects of FA and OZ on combined dietary fat- and fructose-induced metabolic disorders.

Therefore, the aim of this study was to investigate and compare the effects of dietary supplied FA and OZ on high-fat and high-fructose diet (HFFD)-induced MS in rats and to explore their potential mechanisms. FA and OZ were added at equimolar concentration. Lipid and glucose metabolism, IR index, hepatic functions, inflammation status, serum antioxidant capacity, and hepatic histological changes were measured. The mechanisms of their effects were further investigated in oleic acid-stimulated HepG2 cells.

## Materials and Methods

### Animals and diets

Twenty-four male SD rats weighing 350–360 g were purchased from the Experimental Animal Center, Health Science Department of Peking University [Certificate No. SCXK (Beijing) 2013–0001]. Animals were kept in a controlled temperature (23°C ± 2°C) and humidity (55% ± 5%) room on a 12:12-h light/dark cycle and were fed with standard laboratory chow (Experimental Animal Center, Beijing, China) for one week. After adaptation period, rats were randomly divided into four groups (n = 6/group) with equal body weight and were placed on the following diets for 13 weeks: regular rodent diet (NG, 3.2 kcal/g, 4.5% fat, w/w); HFFD (MG, 4.7 kcal/g, 25% fructose and 25% lard); FA group (HFFD plus 0.05% FA, purity 98%); and OZ group (HFFD plus 0.16% OZ, purity 98%). FA and OZ were added at the same molar concentration (2.6 mmol/kg diet). Rats had ad libitum access to food and drinking water. Body weight was measured weekly and food consumption was monitored daily. At the end of the experiment, animals were sacrificed after overnight fasting (12 h). The liver, spleen, kidney, epididymal and perirenal fat pads were dissected and weighed immediately. The protocols were approved by the Ethics Committee of the Beijing Key Laboratory of Functional Food from Plant Resources (Permit number: A330–5) and were conducted in accordance with the guidelines for animal care of the National Institute of Health [18]. All efforts were made to minimize suffering of animals.

### Measurement of biochemical parameters

After a 12-h overnight fasting, blood samples were taken from orbital venous by capillary tube under anesthesia, and centrifuged at 4,000 g for 10 min to obtain serum. The concentrations of serum total triglyceride (TG), total cholesterol (TC), low density lipoprotein-cholesterol (LDL-C), high density lipoprotein-cholesterol (HDL-C), free fatty acid (FFA), total bilirubin (TBIL), and the activities of aspartate transaminase (AST), alanine transaminase (ALT), alkaline phosphatase (ALP), and lactate dehydrogenase (LDH) were measured using corresponding commercial assay kits (Biosid Biotechnology and Science Inc. Beijing, China) on Alcyon 300 automatic analyzer (Alcyon, USA). The total antioxidant capacity (TAOC) and malondialdehyde (MDA) levels of the serum were determined with enzymatic kits purchased from Nanjing Jiancheng Bioengineering Inst. (Nanjing, China). Serum insulin concentration was assayed with a radio-immunological assay kit (Insulin RIA kit, Atom High Tech Co., Ltd., Beijing, China). Concentrations of serum leptin, adiponectin, C-reactive protein (CRP), tumor necrosis factor-α (TNF-α), and interleukin-6 (IL-6) were determined by commercial assay kits from Keyingmei Biotechnology and Science Inc. (Beijing, China). The liver was homogenized and the lipids were extracted as previously described [19]. Hepatic TC and TG levels were determined in the same way as the serum lipid profiles.

### Oral glucose (OGTT) and insulin (ITT) tolerance tests

For OGTT, 12 h overnight fasted rats were orally infused with glucose (2 g/kg body weight). Tail blood was collected at 0 (before glucose infusion), 30, 60, 90, and 120 min (post infusion). Blood glucose levels were measured with a glucometer (Johnson & Johnson Investment Ltd., China) at each time point. For ITT, insulin (0.5 U/kg body weight) was injected intraperitoneally after a 4 h fast, and blood tests were carried out as for the OGTT. Total area under the curve (AUC) was calculated for both OGTT and ITT, and HOMA-IR index was determined as previously described [20].

### Histological analysis

For the oil red O staining, hepatic tissues were frozen in liquid nitrogen, sliced and stained with oil red O solution (0.5 g/100 ml, dissolved in isopropanol). For the hematoxylin-eosin (HE) staining, hepatic tissues were fixed in 10% formalin, embedded in paraffin, sliced and stained. All the sections were examined with BA-9000L microscope (Osaka, Japan). Hepatocellular necrosis, fat vacuoles and lipid droplets were indicated by arrows and marked as “hn”, “fv” and “ld”, respectively.

### Cell culture and treatment

The human hepatocellular carcinoma cell line (HepG2) was purchased from the Cell Culture Center of Peking Union Medical Science (Beijing, China) and cultured in Dulbecco’s modification of eagle’s medium (DMEM) supplemented with 10% fetal bovine serum (Gibco Life Technologies, Grand Island, NY). The cytotoxicity of FA and OZ was conducted based on our previous report [20]. After attachment in a 96 well plate for 24 h, cells were incubated with serum-free DMEM containing 2 mM oleic acid-bovine serum albumin (OA-BSA) [21] and OA-BSA containing FA or OZ for 24 h. Intracellular TG content was determined as previously reported [20]. The results obtained from the cell culture were normalized to intracellular total protein and expressed as percentage of control cells.

### RNA extraction and real-time PCR

Total RNA was isolated from HepG2 cells by RNeasy mini kit (QIAGEN, Germany) according to manufacturer’s protocol. The purity of the RNA extract was analyzed by spectrophotometer, and its concentration by Qubit 2.0 Fluorometer (Invitrogen, USA). Gene expression of glyceraldehyde-3-phosphate dehydrogenase (GAPDH), liver X receptors α (LXRα), fatty acid synthase (FAS), stearoyl coenzyme-A desaturase-1 (SCD-1), acetyl coA carboxylase α (ACCα), acetyl coA carboxylase β (ACCβ), sterol regulatory element binding protein 2 (SREBP-2), and 3-hydroxy-3-methylglutaryl coenzyme A reductase (HMGCR) were analyzed. The sequences of the designed primers and probes are shown in Table 1. One-step real-time quantitative reverse transcription polymerase chain reaction (RT-PCR) detection was performed by SentiFAST kit (Bioline, USA) on LightCycler 480II device (Roche, Germany). The reaction parameters were as follows: 45°C for 10 min and 95°C for 2 min before the 40 cycles; each cycle was conducted at 95°C for 5s and at 60°C for 20s. Data were analyzed with LightCycler 480 Software (Roche, Germany). The relative standard method (relative fold change) was employed to calculate the amount of the target gene [22] with GAPDH expression used as control.

**Table 1:** Primer sequences of genes.

Gene	GenBank numbers	Product length (bp)		Primer sequence (5’-3’)
GAPDH	AF261085.1	126	Forward:	GGTGGTCTCCTCTGACTTCAACA
			Reverse:	GTTGCTGTAGCCAAATTCGTTGT
			Fluorescent probe:	CCTCAACGACCACTTTGTCAAGCTCATT
LXRα	AB307698.1	205	Forward:	AACTGGGCATGATCGAGAAG
			Reverse:	AAGCCGGGTAGCTGTTTAGC
			Fluorescent probe:	CCATCGTCTCTGTGCAGGAGATAGTTGA
FAS	U26644.1	234	Forward:	CCAGAGTCGGAGAACTTGC
			Reverse:	CACGATGGCTTCATAGGTGA
			Fluorescent probe:	TCTAGGTTTGATGCCTCCTTCTTCGGAG
SCD-1	AF097514.1	280	Forward:	CCTCTACTTGGAAGACGACATTCGC
			Reverse:	GCAGCCGAGCTTTGTAAGAGCGGT
			Fluorescent probe:	TCTCTGCTACACTTGGGAGCCCTGTATG
ACCα	NM_198839.1	250	Forward:	CGCAGCAATAAGAATGTTTGG
			Reverse:	TGTCCAGCCAGCCAGTATC
			Fluorescent probe:	CAATTTCAAACATGGTGGTGGCTTTGA
ACCβ	NM_001093.3	124	Forward:	CGTGCAGTTGGTCCAGAGAT
			Reverse:	CACACTTGTCGTAGTGGGCT
			Fluorescent probe:	TCCTGAGAAGATACTTGCGTGTTGAGCA
SREBP-2	NR_103834.1	165	Forward:	GTTTCTCCCACCTCAGTTCC
			Reverse:	TATCAAAGGCTGCTGGATGA
			Fluorescent probe:	TCAGCTGCAACAACAGACGGTAATGATC
HMGCR	BC033692.1	190	Forward:	CCAGAGCAAGCACATTAGCA
			Reverse:	CAGCCAAAGCAGCACATAA
			Fluorescent probe:	CCCTCGATGCTCTTGTTGAATGTCTTGT

### Statistical analysis

Results were presented as means ± SD. The statistical significance of the difference between groups was calculated by Kruskal-Wallis test followed by Dunn’s multiple range post-test. Statistical significance was set at P<0.05. All statistical analyses were performed using Prism 5.0 (GraphPad Software, San Diego, CA).

## Results

### Effects of FA and OZ on body weight, food intake, and organ indices

**Fig 1:**
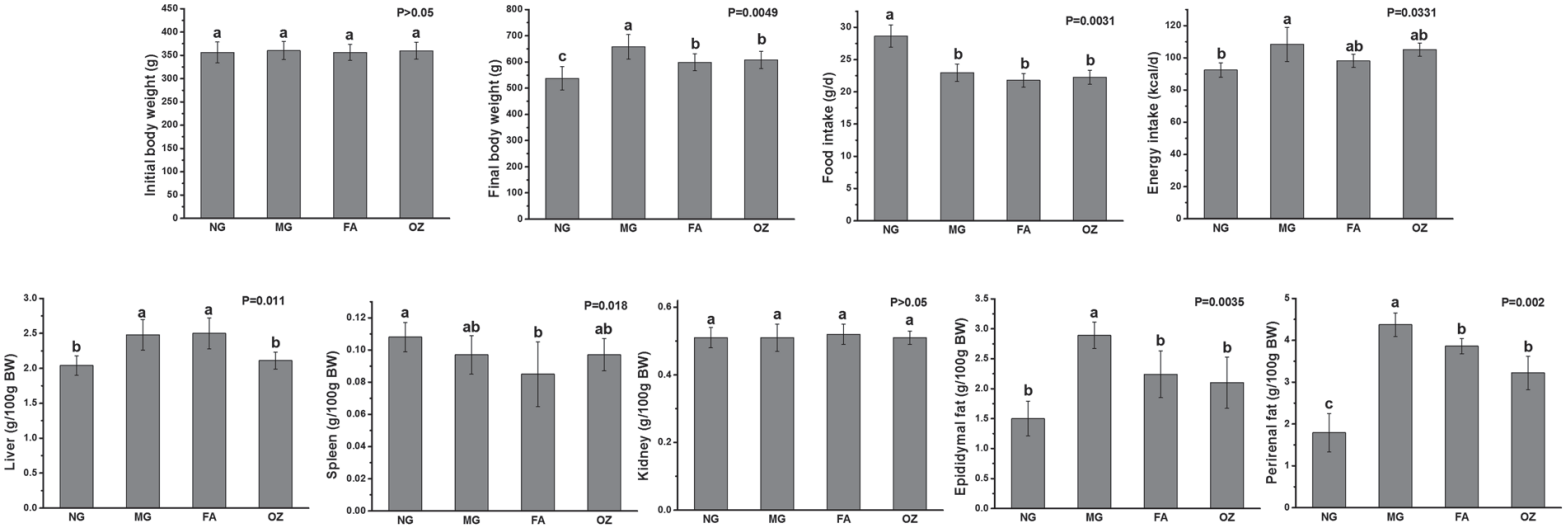
Effects of FA and OZ on body weight, food intake and organ indices. (A): Initial body weight; (B): Final body weight; (C): Food intake; (D): Energy intake; (E): Liver index; (F): Spleen index; (G): Kidney index; (H): Epididymal fat index; (I): Perirenal fat index. Data are presented as mean ± SD (n = 6 per group). Mean values with different letters are significantly different (P<0.05). P values were determined by Kruskal-Wallis test. NG, normal group; MG, high-fat and high-fructose diet (HFFD); FA, HFFD plus ferulic acid; OZ, HFFD plus γ-oryzanol.

HFFD resulted in a significant higher body weight gain in MG compared to NG rats, and this was significantly attenuated by supplementations with FA and OZ (P<0.05, Fig. 1B). Energy intake of HFFD-fed rats was significantly higher compared to that of NG rats (P<0.05), and this was not affected by FA or OZ treatment (Fig. 1D).

Rats fed with HFFD showed significant higher hepatic index and adipose tissue indices compared with NG (P<0.05, Fig. 1E, H and I). The increased hepatic index was markedly reduced by 14.9% in OZ treated group, and the epididymal and perirenal fat indices were significantly decreased by both FA and OZ supplementations (compared with MG, P<0.05). The indices of spleen and kidney were not affected by diet or treatment supplementation (P>0.05, Fig. 1F and G).

### Effects of FA and OZ on serum lipid profiles and hepatic fat accumulation

**Fig 2:**
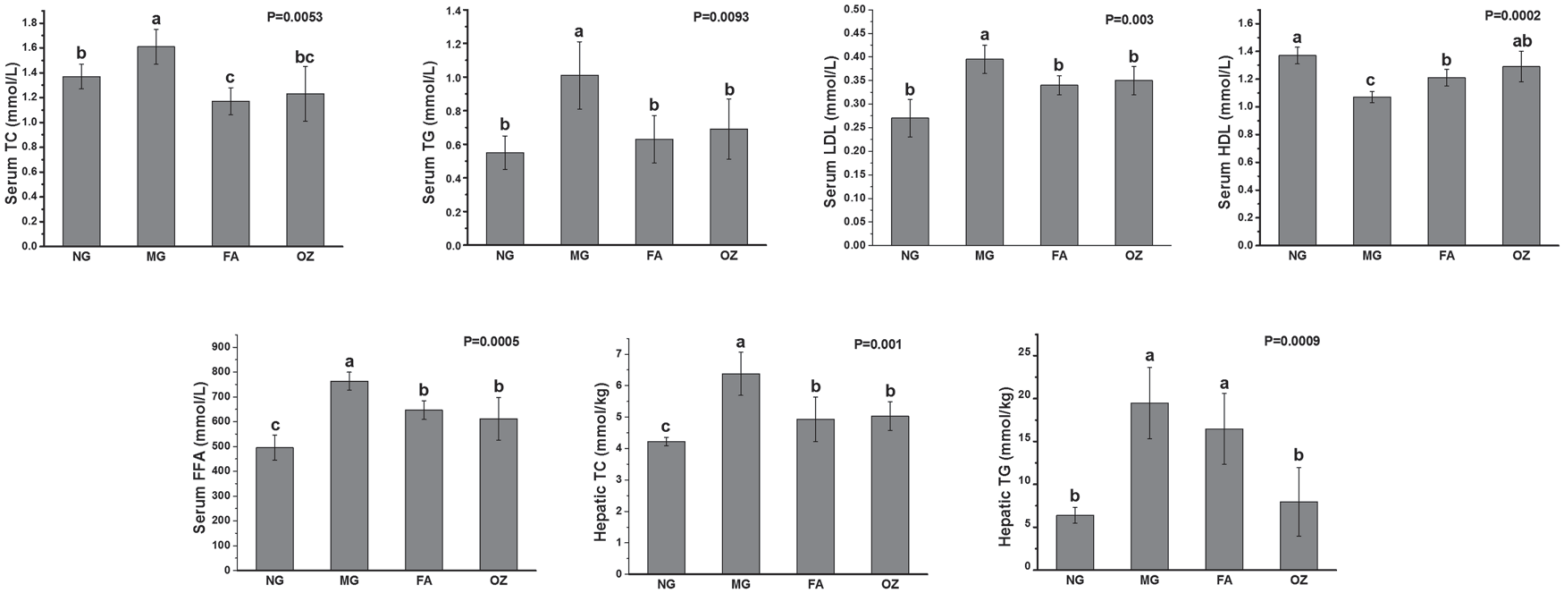
Effects of FA and OZ on serum and hepatic lipid profiles. (A): Serum total cholesterol level; (B): Serum total triglyceride level; (C): Serum low density lipoprotein-cholesterol level; (D): Serum high density lipoprotein-cholesterol level; (E): Serum free fatty acid level; (F): Hepatic total cholesterol level; (G): Hepatic total triglyceride level. Data are presented as mean ± SD (n = 6 per group). Mean values with different letters are significantly different (P<0.05). P values were determined by Kruskal-Wallis test. NG, normal group; MG, high-fat and high-fructose diet (HFFD); FA, HFFD plus ferulic acid; OZ, HFFD plus γ-oryzanol.

As shown in Fig. 2, rats fed with HFFD had significant higher serum TC, TG, LDL-C, FFA levels and lower HDL-C level (compared with NG rats, P<0.05). After administration of FA and OZ, the HFFD induced dyslipidemia was alleviated as serum TG, TC, LDL-C and FFA levels were significantly decreased and the HDL-C levels were markedly increased (compared with MG, P<0.05). OZ was more effective than FA in lowering serum FFA level, but the difference was not statistically significant (P>0.05).

Hepatic lipid profiles were analyzed and presented in Fig. 2. The hepatic TC and TG contents of MG rats were remarkably increased by 1.5 and 3.1 fold, respectively (compared with NG, P<0.05, Fig. 2F and G). After oral administration of FA and OZ, the HFFD-induced increase in hepatic TC content was markedly suppressed by 22.7% and 21.2%, respectively (P<0.05, Fig. 2F). In addition, administration of OZ showed a significant effect on decreasing hepatic TG content by 59.1% (compared with MG, P<0.05, Fig. 2G), while only a decreasing tendency was observed in the FA rats (compared with MG, P>0.05).

### Histopathological analysis

**Fig 3:**
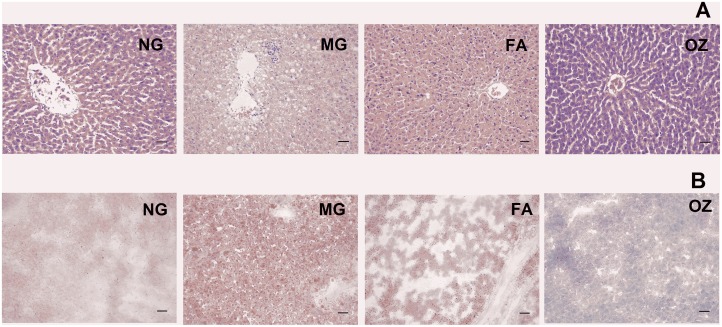
Histological examination of liver. Representative samples of hepatic tissue were stained with hematoxylin and eosin (A) and Oil red O (B). Arrows indicate hepatocellular necrosis, fat vacuoles and lipid droplet were marked as “hn”, “fv” and “ld”, respectively. The bar represents 73 μm. NG, normal group; MG, high-fat and high-fructose diet (HFFD); FA, HFFD plus ferulic acid; OZ, HFFD plus γ-oryzanol.

The histopathological analysis of hepatic sections revealed the presence of TG fat vacuoles and hepatocellular necrosis which were clearly observed in MG rats (Fig. 3A). This condition was markedly improved following FA and OZ treatment, with hepatic tissue returning to its normal state. Similarly, more red dyed fatty droplets were present in MG hepatic sections indicating increased accumulation of lipid under HFFD treatment (Fig. 3B). OZ treatment attenuated HFFD-induced hepatic lipid accumulation and its effect was more pronounced than that of FA treatment. These findings are in agreement with changes in the liver index (Fig. 1E) and hepatic TG content (Fig. 2G).

### Effects of FA and OZ on glucose tolerance, fasting glucose, fasting insulin, and IR

**Fig 4:**
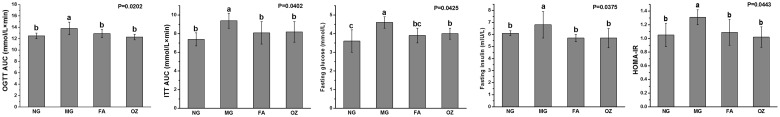
Effects of FA and OZ on serum glucose, insulin, and AUC for OGTT and ITT. (A): AUC of oral glucose tolerance test; (B) AUC of insulin tolerance test; (C): Fasting glucose level;(D): Fasting insulin level; (E): Homeostasis model of assessment for insulin resistance index. Data are presented as mean ± SD (n = 6 per group). Mean values with different letters are significantly different (P<0.05). P values were determined by Kruskal-Wallis test. NG, normal group; MG, high-fat and high-fructose diet (HFFD); FA, HFFD plus ferulic acid; OZ, HFFD plus γ-oryzanol.

After 13-week of HFFD feeding, MG rats had significantly increased fasting glucose and insulin levels compared with NG group (P<0.05, Fig. 4C and D). The AUC for OGTT, ITT as well as the HOMA-IR values were all increased in HFFD-fed rats, indicating impaired glucose tolerance and insulin resistance (P<0.05, Fig. 4A, B and E). Treatment of FA and OZ significantly lowered the AUC for OGTT by 6.5% and 10.9%, respectively (Fig. 4A), and notably improved fasting hyperglycemia by 15.2% and 13.0%, respectively (compared with MG, P<0.05, Fig. 4C). Both treatments improved hyperinsulinemia equally, and markedly reduced AUC of ITT and HOMA-IR values (compared with MG, P<0.05, Fig. 4B, D and E).

### Effects of FA and OZ on hepatic function

**Fig 5:**
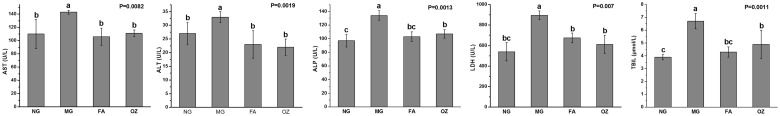
Effects of FA and OZ on hepatic function. (A): Aspartate aminotransferase activity; (B): Alanine aminotransferase activity; (C): Alkaline phosphatase activity; (D): Lactate dehydrogenase activity; (E) Total bilirubin level. Data are presented as mean ± SD (n = 6 per group). Mean values with different letters are significantly different (P<0.05). P values were determined by Kruskal-Wallis test. NG, normal group; MG, high-fat and high-fructose diet (HFFD); FA, HFFD plus ferulic acid; OZ, HFFD plus γ-oryzanol.

The results of serum analysis showed that the activities of serum AST, ALT, ALP, and LDH in MG rats were significantly increased compared to those of NG rats (P<0.05, Fig. 5), and the serum TBIL concentration was also significantly elevated by 1.7-fold (compared with NG, P<0.05, Fig. 5E). In comparison with MG rats, the HFFD- induced hepatic function impairment was significantly alleviated by FA and OZ administration as the activities of serum AST, ALT, ALP and LDH were normalized, and the TBIL levels were markedly lowered by 35.8% and 26.9%, respectively (P<0.05, Fig. 5).

### Effects of FA and OZ on serum antioxidant capacity

**Fig 6:**
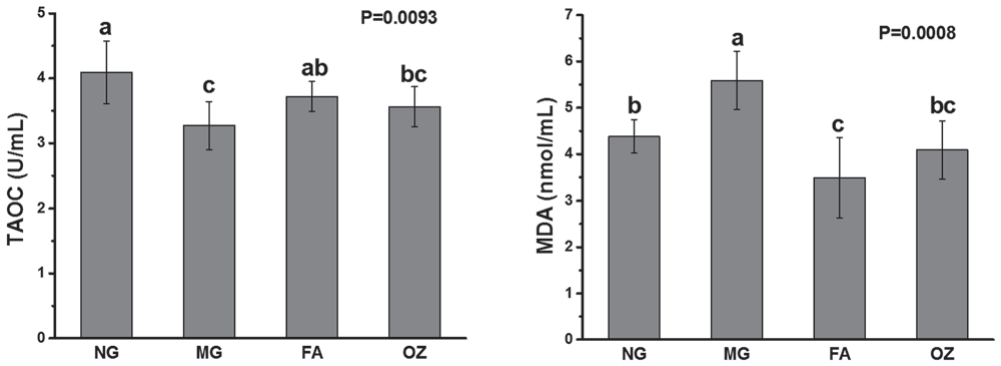
Effects of FA and OZ on serum antioxidant capacity. (A): Total antioxidant capacity, (B): Malondialdehyde level. Data are presented as mean ± SD (n = 6 per group). Mean values with different letters are significantly different (P<0.05). P values were determined by Kruskal-Wallis test. NG, normal group; MG, high-fat and high-fructose diet (HFFD); FA, HFFD plus ferulic acid; OZ, HFFD plus γ-oryzanol.

MG rats showed a significant decrease in serum TAOC and a marked elevation in MDA content (compared with NG, P<0.05, Fig. 6), which indicated a decline in antioxidant status. FA treatment significantly increased the TAOC by 13.8% (compared with MG, P<0.05, Fig. 6A) while OZ supplementation showed a similar tendency but did not reach significance (P>0.05). Compared with MG rats, the serum MDA levels of FA and OZ rats were significantly decreased by 37.6% and 26.8%, respectively (P<0.05, Fig. 6B).

### Effects of FA and OZ on serum cytokines

**Fig 7:**
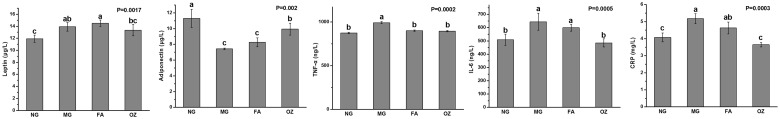
Effects of FA and OZ on serum cytokines levels. (A): Leptin level; (B): Adiponectin level; (C): Tumor necrosis factor α level; (D): Interleukin-6 level; (E): C-reactive protein level. Data are presented as mean ± SD (n = 6 per group). Mean values with different letters are significantly different (P<0.05). P values were determined by Kruskal-Wallis test. NG, normal group; MG, high-fat and high-fructose diet (HFFD); FA, HFFD plus ferulic acid; OZ, HFFD plus γ-oryzanol.

HFFD consumption resulted in a remarkable decline in adiponectin level and marked increases in serum leptin, TNF-α, IL-6, and CRP concentrations (compared with NG, P<0.05, Fig. 7). Compared with MG rats, both FA and OZ treatment significantly reduced TNF-α level (Fig. 7C), and only OZ administration markedly decreased serum CRP and IL-6 levels and increased adiponectin level (P<0.05, Fig. 7A and E). Neither FA nor OZ decreased serum leptin concentration significantly (compared with MG, P>0.05, Fig. 7A).

### Effects of FA and OZ on TG accumulation in HepG2 cells

**Fig 8:**
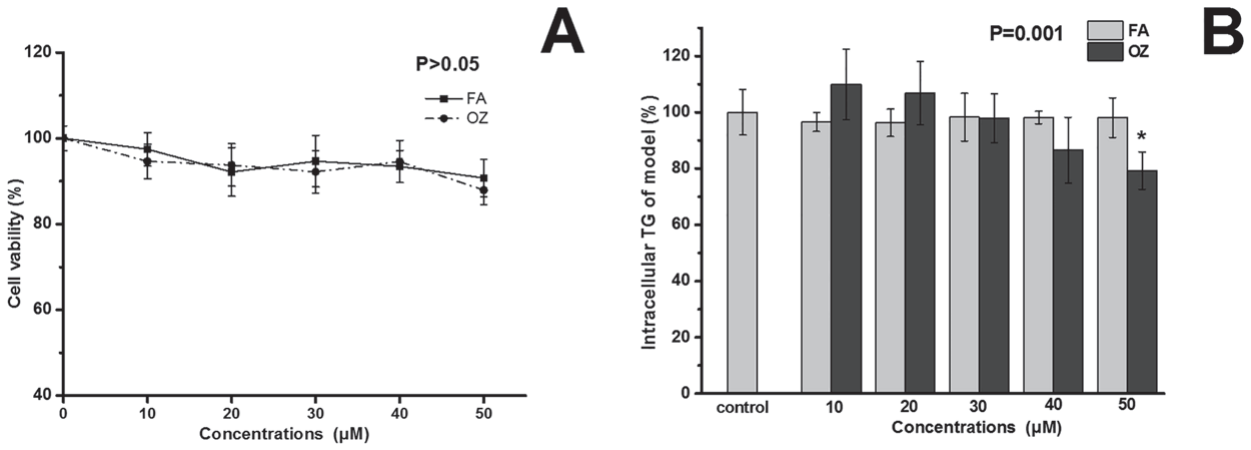
Effects of FA and OZ on cell viability (A) and intracellular TG content (B). The cells in (B) were incubated with serum free DMEM containing 2 mM OA for 24 h. Ferulic acid or γ-oryzanol was added at 50 μM. Data are presented as mean ± SD (n = 8 per group). *P<0.05 compared with control. The P value in (B) was determined by control and 50μM OZ.

MTT assay was conducted to avoid cytotoxicity. Neither FA nor OZ influenced cell viability under the concentrations used (Fig. 8A). OA induction can lead to TG accumulation and IR in HepG2 cells [21]. Therefore, this cell model was used to evaluate the inhibitory effects of FA and OZ on hepatocytes fat deposition. Compared with control group, FA treatment did not regulate intracellular TG content, whereas OZ treatment significantly inhibited intracellular TG accumulation at 50 μM (P<0.05, Fig. 8B).

### Expressions of regulatory genes

**Fig 9:**
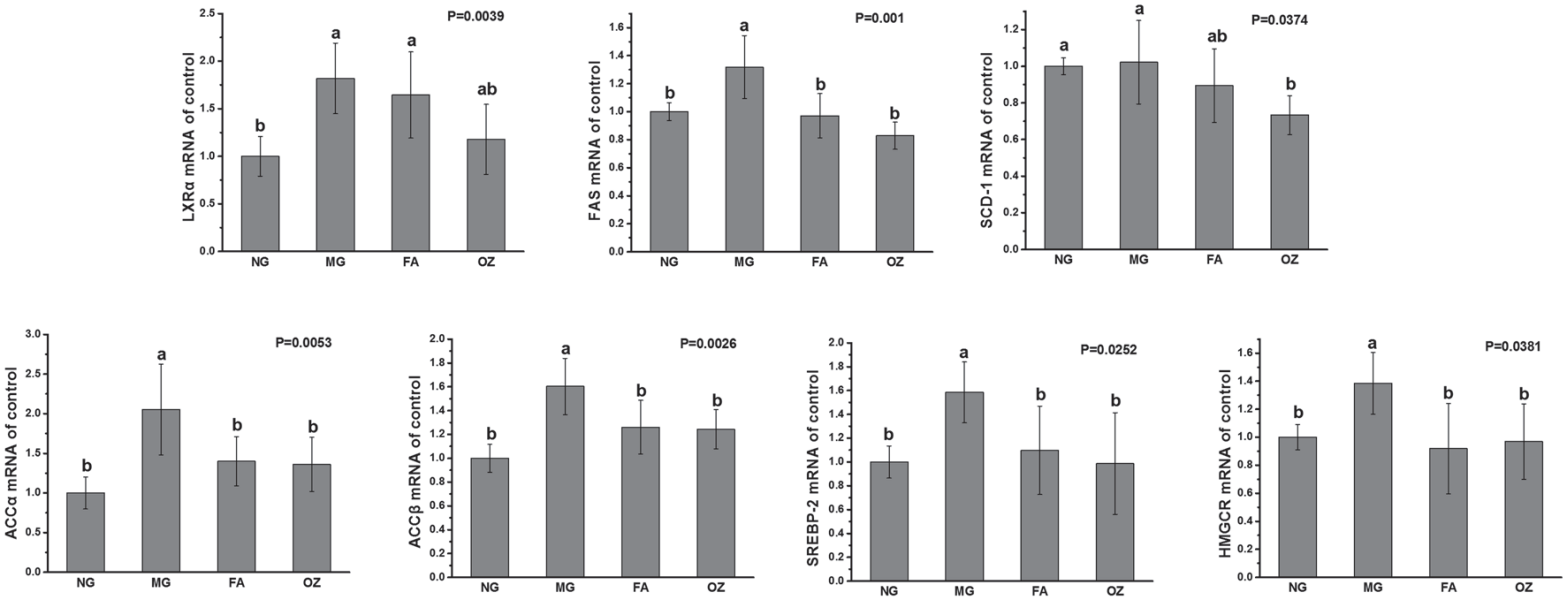
Effects of FA and OZ on gene expressions in OA stimulated HepG2 cell. GAPDH was used for normalization of all target genes. Data are presented as mean ± SD (n = 6 per group) and expressed as fold change over NG. Mean values with different letters are significantly different (P<0.05). P values were determined by Kruskal-Wallis test. NG, normal cells; MG, cells incubated with serum free DMEM containing 2 mM OA; FA, MG treatment containing 50 mM ferulic acid; OZ, MG treatment containing 50 mM γ-oryzanol; GAPDH, glyceraldehyde-3-phosphate dehydrogenase; LXRα, liver X receptors α; FAS, fatty acid synthase; SCD-1, stearoyl coenzyme-A desaturase-1; ACCα, acetyl coA carboxylase α; ACCβ, acetyl coA carboxylase β; SREBP-2, sterol regulatory element binding protein 2; HMGCR, 3-hydroxy-3-methylglutaryl coenzyme A reductase.

As shown in Fig. 9, mRNA expressions of LXRα, FAS, ACCα, ACCβ, SREBP-2, and HMGCR in MG cells were significantly increased than those of NG cells (P<0.05, Fig. 9). Both FA and OZ treatment significantly inhibited gene expressions of FAS, ACCα, ACCβ, SREBP-2, and HMGCR (compared with MG, P<0.05). The inhibitory effects of OZ on LXRα and FAS expressions were slightly better than FA’s, although no statistical significance was noted (P>0.05). Moreover, only OZ significantly down-regulated expression of SCD-1 (compared with MG, P<0.05).

## Discussion

The incidence of MS is influenced by a variety of lifestyle factors with dietary patterns playing a crucial role. Diets rich in fats and fructose have been shown to cause a host of detrimental effects leading to several pathologies and have been frequently used in studies seeking to induce dysfunctions characteristics of metabolic syndrome [23,24]. Indeed, in the present study, feeding a HFFD resulted in increased body weight gain, which was associated with symptoms of MS, such as hyperlipidemia, hyperglycemia, IR and hyperinsulinemia (see Figs. 1, 2, and 4).

FA and OZ, derived from plants, are well-researched natural compounds due to their abundant resources and reported health benefits [13–15]. Supplementations of FA or OZ in HFFD had no effect on daily food intake (Fig. 1C). Since both compounds were added to the diet at equal molar concentrations and given the fact that FA is the major metabolite of OZ [25], it can be reasonably assumed that equal amount of FA might be deposited in the target organs resulting in similar *in vivo* effects.

Our results showed that FA and OZ exhibited similar effects in alleviating HFFD-induced obesity and dyslipidemia (Figs. 1 and 2). Obesity, especially visceral obesity, plays a central role in MS and is a risk factor for other HFFD-induced diseases. As shown in Fig. 1, HFFD caused a significant increase in body weight and adipose tissue mass in MG group. These were significantly diminished by FA and OZ treatment (P<0.05, Fig. 1), demonstrating an anti-obesity effect of dietary supplemented FA and OZ. Moreover, compared with that of MG rats, the serum lipid profile was normalized by FA and OZ treatment as serum TG, TC, LDL-C and FFA levels were significantly decreased and the HDL-C levels were significantly elevated (P<0.05, Fig. 2). These findings are in agreement with previous work showing hypolipidemic effects of FA and OZ treatment in rodents [26, 27], which is largely due to increased fecal lipid excretion [14].

Treatment with FA and OZ also improved parameters of type 2 diabetes. As such, fasting glucose levels were significantly lowered by FA and OZ supplementation (P<0.05, Fig. 4C). When rats were challenged with a glucose load, FA and OZ significantly decreased the AUC values for OGTT (P<0.05, Fig. 4A), suggesting an enhancement in glucose disposal. As FA and OZ inhibit the activities of glucose–6–phosphatase and phosphoenolpyruvate carboxykinase [13], the hepatic gluconeogenesis might be reduced, thus contributing to the alleviation of type 2 diabetic–like parameters. Moreover, exaggerated endoplasmic reticulum stress is also closely related with diabetes [28] and it was ameliorated by FA and OZ treatment [29, 30], indicating another possible pathway by which FA and OZ regulate glucose metabolism.

Consumption of diets rich in fats and fructose can lead to hepatic fat accumulation and liver dysfunctions [23], conditions that are underlined by leakage of cellular enzymes, such as ALT, AST, ALP and LDH [31]. In the present study, the activity of these enzymes and the concentration of TBIL were all normalized by FA and OZ treatment (Fig. 5), demonstrating an attenuation effect on hepatic necrosis, which is probably due to hepatocytes integrity maintenance [32].

Although the pathogenesis of MS has not been clearly deciphered, oxidative stress and IR are two main contributing factors [33]. For example, chronic fructose consumption results in excessive reactive oxygen species, which would cause oxidative damage and insulin signal disruption [34]. The concentration of thiobarbituric acid reactive substances (TBARS) is an indicator of lipid peroxidation process and oxidative stress, and both plasma and erythrocyte lipid TBARS levels could be decreased by FA and OZ treatment [14]. Our results are in aggrement with previous work demonstrating *in vivo* antioxidant capacity following FA and OZ treatment (Fig. 6). It was suggested that OZ prevented *in vivo* oxidation through its ferulate moiety [35], which might account for the similar effects of FA and OZ. Compared with OZ, FA treatment showed better effects in elevating TAOC level and decreasing MDA concentration (Fig. 6), which is probably due to its higher level of serum vitamin E [36]. Moreover, in the present study, the AUC for ITT and HOMA-IR value were significantly decreased by FA and OZ treatment (Fig. 4B and E), suggesting an increase in insulin sensitivity and alleviation of IR in both peripheral and hepatic tissues.

Although some similar *in vivo* effects were observed following FA and OZ administration, there is a significant differential effect in the inhibition of hepatic fat deposition. Compared to FA, OZ treatment resulted in a remarkable lower liver weight (Fig. 1E) and hepatic TG content (Fig. 2G), which was further confirmed by hepatic histological analysis (Fig. 3B). In addition, serum FFA level in OZ rats was lower than that of the FA rats (Fig. 2E), which indicate less active compound required for hepatic TG synthesis and accumulation, since FFA influx stimulates hepatic lipogenesis [37].

To confirm the effect of OZ in the inhibition of hepatic TG accumulation and to investigate possible mechanisms, an *in vitro* study was employed using OA-stimulated HepG2 cells. The results showed that only the OZ-treated cells exhibited significant lower intracellular TG content (Fig. 8B), which is consistent with our *in vivo* results (Fig. 2G). Endogenous lipogenesis is one of the major sources of hepatic lipids [38]. LXRα is the upstream transcription factor that regulates lipogenic enzymes such as FAS [39, 40], and FAS catalyzes the last step of fatty acid biosynthesis [41]. Thus, as shown in Fig. 9, expressions of LXRα and FAS were potently inhibited by OZ treatment, indicating a potential inhibitory effect of OZ on fatty acid *de novo* synthesis. Moreover, the absorbed or synthesized fatty acid is finally converted into TG and stored in hepatocytes. SCD–1 is one of the major enzymes that regulates this process [42] and its expression was significantly reduced by OZ treatment, resulting in lower hepatic TG content in OZ rats (Fig. 9)

Finally, treatment with FA and OZ differentially affected cytokine secretion. Inflammation had been causally related with the incidence of MS [43]. TNF-α, IL-6 and CRP are sensitive markers of inflammation that regulate intermediary metabolism, such as synthesis of FFA [44], hydrolysis of TG [45], and function of pancreatic β-cells [46,47]. The levels of TNF-α, IL-6, and CRP were significantly reduced by OZ treatment, whereas only secretion of TNF-α was controlled by FA (Fig. 7). OZ treatment also significantly improved secretion of adiponectin, which might stimulate glucose utilization and fatty acid oxidation [48] (Fig. 7B). In the present study, although FA and OZ were used at equal molar concentration, the latter exhibited far better anti-inflammatory effects and elevated adiponectin level (Fig. 7). This could probably be due to their metabolites difference as OZ consists of FA esters with phytosterols, such as campesterol, stigmasterol, or β-stigmasterol [11]. The ester bond of OZ is broken down during digestion followed by phytosterols release [49], with subsequent beneficial effects on metabolic dysregulation. It has been shown that daily intake of diet rich in campesterol, stigmasterol, or β-stigmasterol could lower pro-inflammatory cytokine production and increase serum adiponectin level [50–53]. In addition, phytosterols can also regulate TG metabolism [53] and have been recommended as LDL cholesterol-lowering agents [54]. Therefore, the phytosterols produced by OZ digestion might contribute to the greater improvement in metabolic parameters by OZ over FA.

## Conclusion

In summary, OZ and FA exhibit similar effects in alleviating HFFD-induced obesity, hyperlipidemia, hyperglycemia, hepatic injury, and IR. However, the effect of OZ in inhibiting hepatic fat deposition and anti-inflammatory were significantly greater than those of FA, which are possibly due to the down-regulation of lipogenesis genes expression and differences in their metabolites.

## References

[1] Simmons R, Alberti K, Gale E, Colagiuri S, Tuomilehto J, et al (2010) The metabolic syndrome: useful concept or clinical tool? Report of a WHO Expert Consultation. Diabetologia 53: 600–605. 10.1007/s00125-009-1620-4 20012011

[2] Ärnlöv J, Ingelsson E, Sundström J, Lind L (2010) Impact of body mass index and the metabolic syndrome on the risk of cardiovascular disease and death in middle-aged men. Circulation 121: 230–236. 10.1161/CIRCULATIONAHA.109.887521 20038741

[3] Kassi E, Pervanidou P, Kaltsas G, Chrousos G (2011) Metabolic syndrome: definitions and controversies. BMC Med 9: 48 10.1186/1741-7015-9-48 21542944 PMC3115896

[4] Johnson RJ, Segal MS, Sautin Y, Nakagawa T, Feig DI, et al (2007) Potential role of sugar (fructose) in the epidemic of hypertension, obesity and the metabolic syndrome, diabetes, kidney disease, and cardiovascular disease. Am J Clin Nutr 86: 899–906. 17921363 10.1093/ajcn/86.4.899

[5] Yamauchi T, Kamon J, Waki H, Terauchi Y, Kubota N, et al (2001) The fat-derived hormone adiponectin reverses insulin resistance associated with both lipoatrophy and obesity. Nat Med 7: 941–946. 11479627 10.1038/90984

[6] Dills W (1993) Protein fructosylation: fructose and the Maillard reaction. Am J Clin Nutr 58: 779S–787S. 8213610 10.1093/ajcn/58.5.779S

[7] Tappy L, Lê KA (2010) Metabolic effects of fructose and the worldwide increase in obesity. Physiol Rev 90: 23–46. 10.1152/physrev.00019.2009 20086073

[8] Zhang H, Zhou F, Ji B, Li B, Luo Y, et al (2009) Effects of fructose and/or fat in the diet on developing the type 2 diabetic-like syndrome in CD-1 mice. Horm Metab Res 41: 40–45. 10.1055/s-0028-1087187 18825613

[9] Zhao Z, Moghadasian MH (2008) Chemistry, natural sources, dietary intake and pharmacokinetic properties of ferulic acid: a review. Food Chem 109: 691–702. 26049981 10.1016/j.foodchem.2008.02.039

[10] Ardiansyah, Shirakawa H, Koseki T, Ohinata K, Hashizume K, et al (2006) Rice bran fractions improve blood pressure, lipid profile, and glucose metabolism in stroke-prone spontaneously hypertensive rats. J Agric Food Chem 54: 1914–1920. 16506853 10.1021/jf052561l

[11] Ghatak S, Panchal S (2011) Gamma-oryzanol-A multi-purpose steryl ferulate. Current Nutrition & Food Science 7: 10–20. 10.5049/EBP.2014.12.2.80 25606636

[12] Lerma Garcia M, Herrero Martinez J, Simó Alfonso E, Mendonça CR, Ramis Ramos G (2009) Composition, industrial processing and applications of rice bran γ-oryzanol. Food Chem 115: 389–404.

[13] Son MJ, Rico CW, Nam SH, Kang MY (2011) Effect of oryzanol and ferulic acid on the glucose metabolism of mice fed with a high-fat diet. J Food Sci 76: H7–H10. 10.1111/j.1750-3841.2010.01907.x 21535685

[14] Son MJ, Rico CW, Nam SH, Kang MY (2010) Influence of oryzanol and ferulic acid on the lipid metabolism and antioxidative status in high fat-fed mice. J Clin Biochem Nutr 46: 150–156. 10.3164/jcbn.09-98 20216948 PMC2831094

[15] Chotimarkorn C, Ushio H (2008) The effect of *trans-*ferulic acid and gamma-oryzanol on ethanol-induced liver injury in C57BL mouse. Phytomedicine 15: 951–958. 10.1016/j.phymed.2008.02.014 18424018

[16] Alwahsh SM, Xu M, Schultze FC, Wilting J, Mihm S, et al (2014) Combination of alcohol and fructose exacerbates metabolic imbalance in terms of hepatic damage, dyslipidemia, and insulin resistance in rats. Plos One 9: e104220 10.1371/journal.pone.0104220 25101998 PMC4125190

[17] Sloboda DM, Li M, Patel R, Clayton ZE, Yap C, et al (2014) Early Life Exposure to Fructose and Offspring Phenotype: Implications for Long Term Metabolic Homeostasis. J Obesity 2014. 10.1155/2014/203474 PMC4017842 24864200

[18] National Research Council (1985) Guide for the care and use of laboratory animals NIH publication No. 85. Bethesda, MD: National Institutes of Health: 23.

[19] Folch J, Lees M, Sloane Stanley G (1957) A simple method for the isolation and purification of total lipids from animal tissues. J Biol Chem 226: 497–509. 13428781

[20] Liu J, Zhang H, Ji B, Cai S, Wang R, et al (2014) A diet formula of Puerariae radix, Lycium barbarum, Crataegus pinnatifida, and Polygonati rhizoma alleviates insulin resistance and hepatic steatosis in CD-1 mice and HepG2 cells. Food Funct 5: 1038–1049. 10.1039/c3fo60524h 24626737

[21] Vidyashankar S, Sandeep Varma R, Patki PS (2013) Quercetin ameliorate insulin resistance and up-regulates cellular antioxidants during oleic acid induced hepatic steatosis in HepG2 cells. Toxicol in Vitro 27: 945–953. 10.1016/j.tiv.2013.01.014 23348005

[22] Pfaffl MW (2001) A new mathematical model for relative quantification in real-time RT-PCR. Nucleic Acids Res 29: 2003–2007. 11328886 10.1093/nar/29.9.e45 PMC55695

[23] Panchal SK, Poudyal H, Iyer A, Nazer R, Alam A, et al (2011) High-carbohydrate high-fat diet-induced metabolic syndrome and cardiovascular remodeling in rats. Journal Cardiovasc Pharm 57: 51–64. 10.1097/FJC.0b013e3181feb90a 21572266

[24] Panchal SK, Brown L (2010) Rodent models for metabolic syndrome research. J Biomed Biotechnol 2011: 1–14. 10.1155/2011/351982 PMC3018657 21253582

[25] Fujiwara S, Sakurai S, Noumi K, Sugimoto I, Awata N (1980) Metabolism of gamma-oryzanol in rabbit (author’s transl, article in Japanese). Yakugaku zasshi 100: 1011 7218141

[26] Wilson TA, Nicolosi RJ, Woolfrey B, Kritchevsky D (2007) Rice bran oil and oryzanol reduce plasma lipid and lipoprotein cholesterol concentrations and aortic cholesterol ester accumulation to a greater extent than ferulic acid in hypercholesterolemic hamsters. J Nutr Biochem 18: 105–112. 16713234 10.1016/j.jnutbio.2006.03.006

[27] Kim HK, Jeong TS, Lee MK, Park YB, Choi MS (2003) Lipid-lowering efficacy of hesperetin metabolites in high-cholesterol fed rats. Clin Chim Acta 327: 129–137. 12482628 10.1016/s0009-8981(02)00344-3

[28] Özcan U, Cao Q, Yilmaz E, Lee A-H, Iwakoshi NN, et al (2004) Endoplasmic reticulum stress links obesity, insulin action, and type 2 diabetes. Science 306: 457–461. 15486293 10.1126/science.1103160

[29] Hiratsuka T, Matsuzaki S, Miyata S, Kinoshita M, Kakehi K, et al (2010) Yokukansan inhibits neuronal death during ER stress by regulating the unfolded protein response. Plos One 5: e13280 10.1371/journal.pone.0013280 20967273 PMC2953506

[30] Kozuka C, Yabiku K, Sunagawa S, Ueda R, Taira SI, et al (2012) Brown rice and its component, γ-oryzanol, attenuate the preference for high-fat diet by decreasing hypothalamic endoplasmic reticulum stress in mice. Diabetes 61: 3084–3093. 10.2337/db11-1767 22826028 PMC3501875

[31] Poudyal H, Campbell F, Brown L (2010) Olive leaf extract attenuates cardiac, hepatic, and metabolic changes in high carbohydrate-, high fat-fed rats. J Nutr 140: 946–953. 10.3945/jn.109.117812 20335636

[32] Srinivasan M, Sudheer AR, Menon VP (2007) Ferulic acid: therapeutic potential through its antioxidant property. J Clinical Biochem Nutr 40: 92–100. 10.3164/jcbn.40.92 18188410 PMC2127228

[33] Lomonaco R, Ortiz-Lopez C, Orsak B, Webb A, Hardies J, et al (2012) Effect of adipose tissue insulin resistance on metabolic parameters and liver histology in obese patients with nonalcoholic fatty liver disease. Hepatology 55: 1389–1397. 10.1002/hep.25539 22183689

[34] Houstis N, Rosen ED, Lander ES (2006) Reactive oxygen species have a causal role in multiple forms of insulin resistance. Nature 440: 944–948. 16612386 10.1038/nature04634

[35] Berger A, Rein D, Schäfer A, Monnard I, Gremaud G, et al (2005) Similar cholesterol-lowering properties of rice bran oil, with varied γ-oryzanol, in mildly hypercholesterolemic men. Eur J Nutr 44: 163–173. 15309429 10.1007/s00394-004-0508-9

[36] Saltiel AR, Kahn CR (2001) Insulin signalling and the regulation of glucose and lipid metabolism. Nature 414: 799–806. 11742412 10.1038/414799a

[37] Marra F, Gastaldelli A, Svegliati Baroni G, Tell G, Tiribelli C (2008) Molecular basis and mechanisms of progression of non-alcoholic steatohepatitis. Trends Mol Med 14: 72–81. 10.1016/j.molmed.2007.12.003 18218340

[38] Cohen JC, Horton JD, Hobbs HH (2011) Human fatty liver disease: old questions and new insights. Science 332: 1519–1523. 10.1126/science.1204265 21700865 PMC3229276

[39] Postic C, Girard J (2008) The role of the lipogenic pathway in the development of hepatic steatosis. Diabetes Metab 34: 643–648. 10.1016/S1262-3636(08)74599-3 19195625

[40] Currie E, Schulze A, Zechner R, Walther TC, Farese RV Jr (2013) Cellular fatty acid metabolism and cancer. Cell Metab 18: 153–161. 10.1016/j.cmet.2013.05.017 23791484 PMC3742569

[41] Li X, Li Z, Xue M, Ou Z, Liu M, et al (2013) Fructus Xanthii attenuates hepatic steatosis in rats fed on high-fat diet. Plos One 8: e61499 10.1371/journal.pone.0061499 23585904 PMC3621865

[42] Pan ZX, Han CX, Wang JW, Li L, Tang H, et al (2011) Cloning and expression of stearoyl-CoA desaturase 1 (SCD-1) in the liver of the Sichuan white goose and landes goose responding to overfeeding. Mol Biology Rep 38: 3417–3425. 10.1007/s11033-010-0451-1 21088902

[43] Chen SJ, Yen CH, Huang YC, Lee BJ, Hsia S, et al (2012) Relationships between inflammation, adiponectin, and oxidative stress in metabolic syndrome. Plos One 7: e45693 10.1371/journal.pone.0045693 23029185 PMC3446937

[44] Grunfeld C, Soued M, Adi S, Moser AH, Dinarello CA, et al (1990) Evidence for two classes of cytokines that stimulate hepatic lipogenesis: relationships among tumor necrosis factor, interleukin-1 and interferon-alpha. Endocrinology 127: 46–54. 1972922 10.1210/endo-127-1-46

[45] Popa C, Netea MG, Van Riel PL, van der Meer JW, Stalenhoef AF (2007) The role of TNF-α in chronic inflammatory conditions, intermediary metabolism, and cardiovascular risk. J Lipid Res 48: 751–762. 17202130 10.1194/jlr.R600021-JLR200

[46] Frühbeck G, Gómez Ambrosi J, Muruzábal FJ, Burrell MA (2001) The adipocyte: a model for integration of endocrine and metabolic signaling in energy metabolism regulation. American Am J Physiol-Endoc M 280: e827–e847. 11350765 10.1152/ajpendo.2001.280.6.E827

[47] Wijesekara N, Krishnamurthy M, Bhattacharjee A, Suhail A, Sweeney G, et al (2010) Adiponectin-induced ERK and Akt phosphorylation protects against pancreatic beta cell apoptosis and increases insulin gene expression and secretion. J Biol Chem 285: 33623–33631. 10.1074/jbc.M109.085084 20709750 PMC2962460

[48] Yamauchi T, Kamon J, Minokoshi Ya, Ito Y, Waki H, et al (2002) Adiponectin stimulates glucose utilization and fatty-acid oxidation by activating AMP-activated protein kinase. Nat Med 8: 1288–1295. 12368907 10.1038/nm788

[49] Huang CJ (2003) Potential functionality and digestibility of oryzanol as determined using in vitro cell culture models. M.Sc. Thesis, Louisiana State University. Available: http://202.28.199.34/multim/3098073.pdf. Accessed 2014 June 17.

[50] Nashed B, Yeganeh B, HayGlass KT, Moghadasian MH (2005) Antiatherogenic effects of dietary plant sterols are associated with inhibition of proinflammatory cytokine production in Apo E-KO mice. J Nutr 135: 2438–2444. 16177209 10.1093/jn/135.10.2438

[51] Micallef MA, Garg ML (2009) Anti-inflammatory and cardioprotective effects of n-3 polyunsaturated fatty acids and plant sterols in hyperlipidemic individuals. Atherosclerosis 204: 476–482. 10.1016/j.atherosclerosis.2008.09.020 18977480

[52] Othman RA, Moghadasian MH (2011) Beyond cholesterol-lowering effects of plant sterols: clinical and experimental evidence of anti-inflammatory properties. Nutr Rev 69: 371–382. 10.1111/j.1753-4887.2011.00399.x 21729090

[53] Baumgartner S, Mensink RP, Plat J (2011) Plant sterols and stanols in the treatment of dyslipidemia: new insights into targets and mechanisms related to cardiovascular risk. Current Pharm Design 17: 922–932. 21418032 10.2174/138161211795428795

[54] Silbernagel G, Genser B, Nestel P, März W (2013) Plant sterols and atherosclerosis. Curr Opin Lipidol 24: 12–17. 10.1097/MOL.0b013e32835b6271 23165086

